# Patients with adrenal insufficiency have cardiovascular features associated with hypovolemia

**DOI:** 10.1007/s12020-020-02458-3

**Published:** 2020-08-19

**Authors:** Daniela Esposito, Emanuele Bobbio, Rosa Di Fraia, Pasquale Mone, Giacomo Accardo, Annamaria De Bellis, Sergio Iorio, Katherine Esposito, Raffaele Marfella, Gudmundur Johannsson, Oskar Ragnarsson, Daniela Pasquali

**Affiliations:** 1 Department of Internal Medicine and Clinical Nutrition, Institute of Medicine, Sahlgrenska Academy, University of Gothenburg, Gothenburg, Sweden; 2 Department of Endocrinology, Sahlgrenska University Hospital, Gothenburg, Sweden; 3 Department of Advanced Medical and Surgical Sciences, University of Campania “Luigi Vanvitelli”, Naples, Italy; 4 Department of Cardiology, Sahlgrenska University Hospital, Gothenburg, Sweden; 5 Department of Molecular and Clinical Medicine, Institute of Medicine at Sahlgrenska Academy, University of Gothenburg, Gothenburg, Sweden

**Keywords:** Adrenal insufficiency, Cardiovascular system, Echocardiography, Blood pressure profile, Modified-release hydrocortisone

## Abstract

**Context:**

Patients with adrenal insufficiency (AI) have excess mortality and morbidity, mainly due to cardiovascular (CV) diseases. The mechanisms for this is unclear.

**Objective:**

To assess CV structure and function in AI patients on conventional replacement therapy and after switching to once-daily, modified-release hydrocortisone (OD-HC) in comparison with healthy matched controls.

**Methods:**

This was a retrospective analysis of 17 adult AI patients (11 with primary AI, 6 with secondary AI) on stable replacement with cortisone acetate [median (minimum, maximum) 33.5 (12.5–50) mg] and, if needed, fludrocortisone [0.1 (0.05–0.2) mg], and 17 healthy matched controls. Ten patients were switched to an equivalent dose of OD-HC. Data from echocardiography, 24 h Holter-ECG and 24 h blood pressure monitoring were collected at baseline and 6 months after the switch to OD-HC.

**Results:**

At baseline, AI patients had smaller left ventricular diastolic diameter (47.1 ± 4.2 vs. 51.6 ± 2.3 mm; *P* = 0.001) and left atrial diameter (34.9 ± 4.7 vs. 38.2 ± 2.6 cm; *P* = 0.018), and a higher ejection fraction (62.5 ± 6.9% vs. 56.0 ± 4.7%; *P* = 0.003) than controls. AI patients had lower nocturnal systolic and diastolic blood pressure than controls (108 ± 15 mmHg vs. 117 ± 8 mmHg; *P* = 0.038 and 65 ± 9 mmHg vs. 73 ± 7 mmHg; *P* = 0.008, respectively). After the switch to OD-HC, nocturnal diastolic blood pressure normalised. No significant changes were observed in echocardiographic and Holter-ECG parameters following the switch.

**Conclusions:**

AI patients on conventional treatment display cardiovascular abnormalities that could be related to hypovolemia. Switch to OD-HC seems to have beneficial effect on blood pressure profile, but no effect on cardiovascular structure and function.

## Introduction

Adrenal insufficiency (AI) is a life-threatening disease characterised by impaired secretion of glucocorticoids (GC), with or without mineralocorticoid (MC) deficiency [1]. The most commonly used replacement regimen consists of hydrocortisone or cortisone acetate administered twice or thrice daily [2, 3]. In patients with primary AI, MC substitution with fludrocortisone is also needed [4].

Long-term outcome in patients with AI was for a long time considered to be similar to that of the general population [5, 6]. However, recent studies have shown that patients with both primary and secondary AI present higher morbidity and mortality compared to the background population, mainly due to cardiovascular (CV) diseases [7–11].

Increased CV morbidity and mortality in patients with AI have been traditionally related to higher cortisol exposure and a GC replacement therapy that fails to mimic the physiological diurnal variation in serum cortisol. Inadequate GC replacement has been associated to adverse lipid profile, increased body weight and diabetes mellitus [10, 12–14]. Available data suggest that patients with AI on conventional GC replacement therapy have an increased prevalence of metabolic syndrome and increased proinflammatory and proatherogenic biomarkers related to an adverse CV risk profile [11]. Novel preparations have been developed aiming to better mimic the circadian cortisol rhythm that could improve the cardio-metabolic outcome [3, 15, 16]. It is important to note that MCs also play a pivotal role in the regulation of circulatory homoeostasis; therefore, impairment of MC replacement in primary AI should be considered as a potential risk factor for poor CV outcomes as well [17].

It is well known that CG and MC receptors are widely expressed in the circulatory system. However, the effects of either adrenal steroid deficiency (e.g. from disease onset until diagnosis and/or under-treatment during stress-related situation) or cortisol overexposure on CV system have not been fully elucidated. In addition, no study has so far investigated whether switching to the novel replacement treatment with once daily modified-release hydrocortisone (OD-HC) may affect structure and function of the CV system.

In this case-control study, we aimed to assess echocardiographic and hemodynamic parameters in patients with AI on stable conventional GC replacement therapy. The secondary aim was to investigate the impact of a switch from conventional GC replacement to OD-HC on the CV system.

## Patients and methods

### Population

**Fig. 1:**
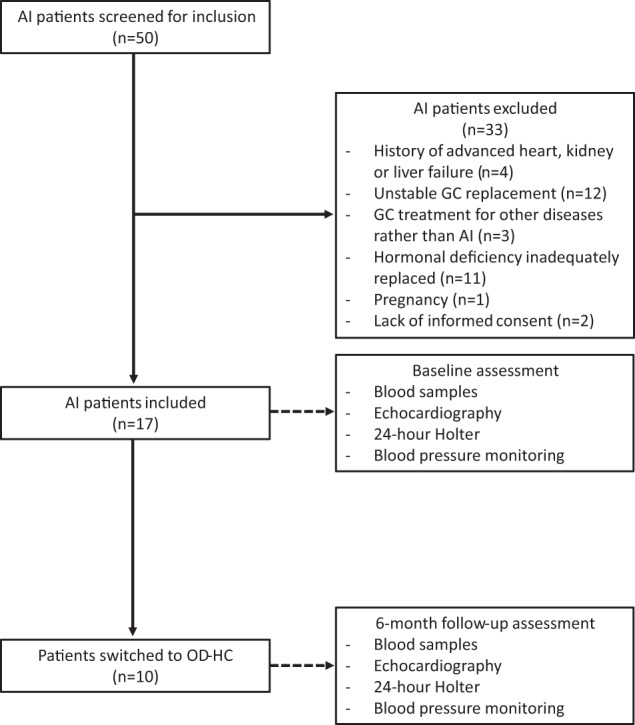
Flow-chart showing enrolment of patients included in the study. A total of 50 consecutive patients with adrenal insufficiency were screened and 17 were included in the study. Of those, 10 patients were switched from conventional therapy with cortisone acetate to once-daily modified-release hydrocortisone. AI adrenal insufficiency

This was a retrospective analysis on data collected from patients with primary and secondary AI followed at the Endocrinology Unit at University of Campania “Luigi Vanvitelli” in Naples between January 28, 2015 and July 28, 2016. Fifty patients with AI were screened and 17 (13 women, 4 men) were included in the study (Fig. 1), with 11 having primary and 6 secondary AI. Diagnosis was made on the basis of clinical evidence and confirmed by cosyntropin stimulation test (short Synacthen test) [18]. Eligibility requirements included age between 18 and 65 years, a diagnosis of primary or secondary AI, and a stable replacement therapy with cortisone acetate for at least 6 months. All patients with primary AI also received MC substitution with fludrocortisone [4]. Patients with secondary AI and other associated hormone deficiencies needed to have a stable replacement with L-thyroxine, testosterone, oestrogen and growth hormone for at least 3 months in order to be eligible for the study. No patient had diabetes insipidus.

Exclusion criteria were: 1. Clinical or laboratory signs of advanced cerebral, respiratory, hepato-biliary or pancreatic diseases; 2. Any other hormonal deficiencies inadequately replaced; 3. Clinically significant renal dysfunction (creatinine level ≥ 150 mmol/l); 4. Active malignancies; 5. Pregnancy; 6. Any other disease requiring medical therapy with GCs. Seventeen healthy subjects among hospital employees, matched for age, gender, smoking habits and body mass index (BMI) served as controls. The study protocol conformed to the ethical guidelines of the Declaration of Helsinki and was approved by the Ethics Committee of the Second University of Naples.

### Study design

Anthropometric variables including weight, height, arterial blood pressure and heart rate (HR) were collected from a baseline visit for all patients and controls. In addition, data on echocardiography, 24 h Holter recording (Holter-ECG), and 24 h ambulatory blood pressure monitoring (BPM) were collected from a baseline visit in both groups. Out of 17 patients with AI, 10 (7 patients with primary and 3 with secondary AI) switched to an equivalent dose of OD-HC (Fig. 1) during the study period. The same clinical data were collected from a follow-up visit after a 6-month period on stable treatment with OD-HC (Fig. 1).

The echocardiography, Holter-ECG and BPM were reviewed by a central core laboratory at the Cardiology Department under the supervision of one of the authors (R.M.).

### Doppler echocardiography

Transthoracic echocardiograms were performed with a two dimensional transthoracic echocardiography (Philips IE 33, Eindhoven, The Netherlands), phased-array transducers at frequencies of 7–12 MHz and analysed for surrogate markers of CV disease, including left atrial diameter (LAD), interventricular septum thickness at diastole (IVSTDd), left ventricle end-diastolic diameter (LVEDd), and left ventricular ejection fraction (LVEF). The presence of valvular disease was assessed according to recommendations of the American Society of Echocardiography [19].

### 24 h Holter-ECG and blood pressure monitoring

A 24 h Holter-ECG recording was performed with an Evo device (Spacelabs Healthcare). During this examination, the patients followed their usual daily routines and activities. The following parameters were recorded in both groups: mean daily and nocturnal HR [HR day and HR night], presence of supraventricular tachycardia (SVT), ventricular premature complexes (VPCs), and presence of ventricular tachycardia (VT).

The blood pressure was monitored using an ambulatory BPM provided by Omron Electronics S.p.A. (Sphygmomanometer Bracelet Intelli Wrap M-L Hem-FL31-E). In this study, 8:00–23:00 was set as daytime, and 23:00–8:00 was set as night-time. The blood pressure was monitored once every 30 min during the day and night for at least 23 h and interpreted according to authoritative literature [20]. The mean systolic and diastolic blood pressure during day- and night-time were calculated.

### Statistical analysis

Data are presented as means and standard deviations (±SD), median (range), numbers and percentages. Statistical analyses were performed with SPSS 25.0 statistical software packages (SPSS Inc. Chicago, IL). Comparisons between groups were performed with unpaired *t*-tests for normally distributed data and the Mann–Whitney U test for non-parametric data. Comparisons between related samples were performed with the Wilcoxon Signed Rank test. All tests were two-tailed and *P* values < 0.05 were considered statistically significant.

Sample size was assessed based on a previous case-control study that analysed echocardiographic characteristics in seven patients with primary AI, before and after GC replacement treatment, and ten healthy controls [21]. To our knowledge, this is the only published study that has investigated echocardiographic parameters in patients with primary AI so far. Using the data on the left ventricular diastolic diameter before and after GC replacement treatment, the inclusion of 16 subjects in two parallel groups of 8 subjects each was needed to yield 80% power with a 5% significance. Since the study design of Fallo et al. [21] was to some extent different from our study, we included a higher number of patients in both groups.

## Results

### Patient characteristics

A total of 17 patients with AI (13 women, 4 men) and 17 matched healthy controls were included in the study. Clinical and demographic characteristics of the study population are presented in Table 1. The mean (±SD) age was 48.5 ± 11.6 years and 49.9 ± 10.8 for patients and controls, respectively (*P* = 0.727) (Table 1). The median (range) daily dose of cortisone acetate was 33.5 (12.5–50) mg, with 14 patients using a twice-daily regimen. The median daily dose of fludrocortisone in patients with primary AI was 0.1 (0.05–0.2) mg.

**Table 1:** Clinical and biochemical characteristics of patients with adrenal insufficiency and matched healthy controls

	AI patients (*n* = 17)	Controls (*n* = 17)	*P*
Age (years)	48.5 ± 11.6	49.9 ± 10.8	0.727
BMI (kg/m^2^)	25.7 ± 3.8	26.0 ± 3.6	0.833
Hypertension (%)	0	0	–
Diabetes mellitus (%)	1 (6)	1 (6)	–
Glycaemia (mg/dL)	84 ± 21	89 ± 14	0.409
Total cholesterol (mg/dL)	217 ± 45	191 ± 22	0.039
LDL-cholesterol (mg/dL)	134 ± 39	144 ± 15	0.346
Triglycerides (mg/dL)	141 ± 64	156 ± 29	0.394
s-Na (mmol/L)	138.8 ± 3.5	138.6 ± 3.3	0.882
s-K (mmol/L)	4.6 ± 1.6	4.2 ± 0.4	0.397

Data are presented in mean (S.D.) or *n* (%)

*AI* adrenal insufficiency, *BMI* body mass index, *s-Na* serum Natrium level, *s-K* serum Kalium level

One patient and one control had type 2 diabetes mellitus and were on treatment with oral hypoglycaemic agents. Neither patients nor controls were receiving antihypertensive treatment. Total serum cholesterol levels were significantly higher in patients than controls (217 ± 45 vs. 191 ± 22, *P* = 0.039) whereas no differences were observed in triglyceride levels. Electrolyte levels as well as fasting plasma glucose did not differ between the groups (Table 1).

### Echocardiography and hemodynamics

**Fig. 2:**
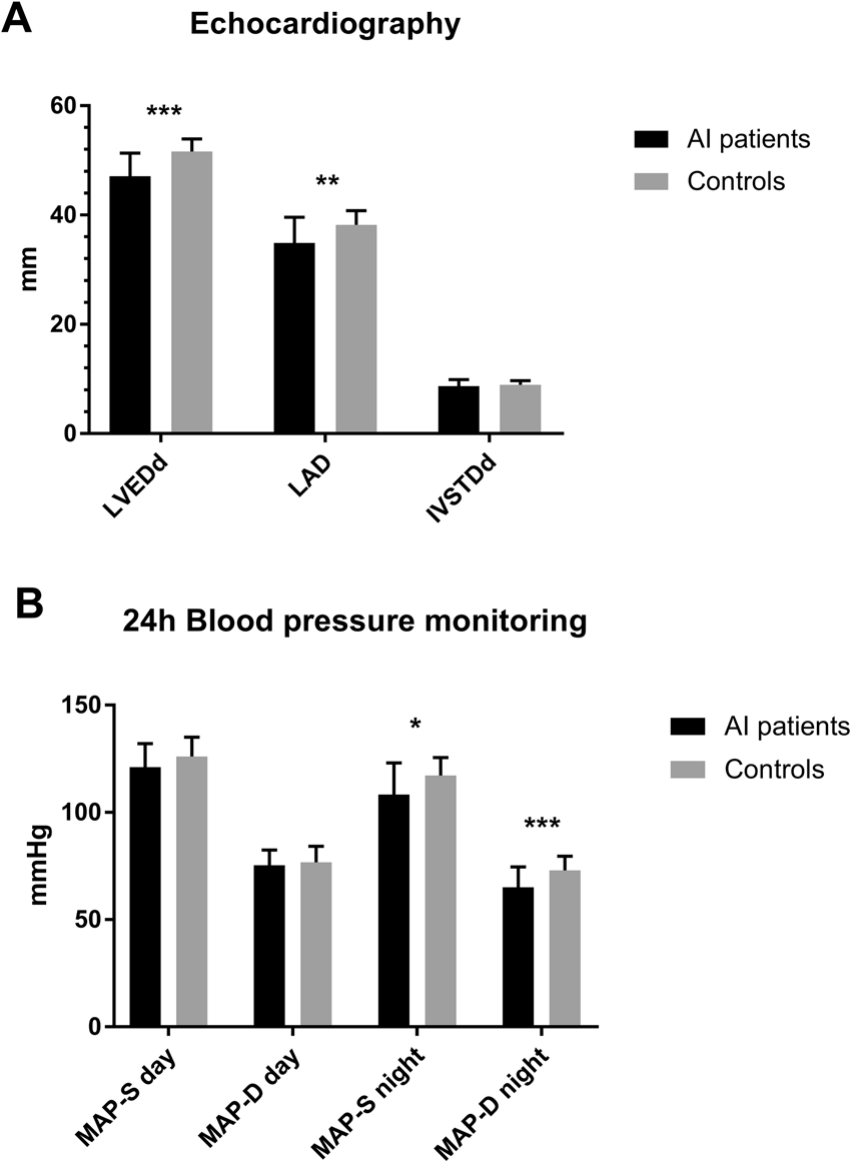
Echocardiographic **a** and 24 h ambulatory blood pressure monitoring findings **b** in patients with adrenal insufficiency on conventional replacement and matched healthy controls. AI adrenal insufficiency, IVSTDd interventricular septum thickness at diastole, LAD Left atrial diameter, LVDEDd left ventricle end-diastolic diameter, MAP-S mean systolic blood pressure, MAP-D mean diastolic blood pressure. ***, *P* < 0.01; **, *P* < 0.02; *, *P* < 0.05

Echocardiographic and hemodynamic parameters are shown in Table 2. Normal left ventricular architecture and function were recorded in the two study groups. However, patients showed a significantly smaller LVEDd (47.1 ± 4.2 mm vs. 51.6 ± 2.3 mm, *P* = 0.001) as well as smaller LAD (34.9 ± 4.7 mm vs. 38.2 ± 2.6 mm, *P* = 0.018) compared with healthy subjects (Fig. 2a). In the sub-groups of patients with primary AI (*n* = 11) and secondary AI (*n* = 6), median LVEDd was 45.9 (42.1–53.0) mm vs. 49.8 (43.3–56.0) mm and LAD was 34.0 (30.0–37.0) mm vs. 38.0 (23.0–44.0) mm, respectively (Table 3). Furthermore, LVEF was significantly higher in patients than in control subjects (62.5 ± 6.9% vs. 56.0 ± 4.7%, *P* = 0.003) (Table 2). Neither group had any evidence of significant valvular disease (Table 2). When the sub-group of patients with primary AI (*n* = 11) were compared to a sub-group of matched controls (*n* = 11), same findings were recorded (Supplementary table 1).

**Table 2:** Echocardiographic, 24 h-blood pressure monitoring and 24 h Holter-ECG findings in patients with adrenal insufficiency and matched healthy controls

	AI patients (*n* = 17)	Controls (*n* = 17)	*P*
Echocardiography			
IVSTDd (mm)	8.7 ± 1.2	8.9 ± 0.8	0.594
LVEDd (mm)	47.1 ± 4.2	51.6 ± 2.3	0.001
LAD (mm)	34.9 ± 4.7	38.2 ± 2.6	0.018
LVEF (%)	62.5 ± 6.9	56.0 ± 4.7	0.003
Patients with significant valvular disease (%)	0	0	–
24 h-blood pressure			
MAP-S day (mmHg)	121 ± 11	126 ± 9	0.155
MAP-D day (mmHg)	75 ± 7	77 ± 8	0.577
MAP-S night (mmHg)	108 ± 15	117 ± 8	0.038
MAP-D night (mmHg)	65 ± 9	73 ± 7	0.008
Holter-ECG			
HR day (bpm)	80 ± 9	74 ± 9	0.069
HR night (bpm)	72 ± 9	68 ± 9	0.259
Patients with SVT (%)	2 (11.8%)	2 (11.8%)	–
VPCs	16 ± 7	7 ± 10	0.567

Data are presented in mean (S.D.) or *n* (%). No cases of supraventricular tachycardia and ventricular tachycardia were recorded

*AI* adrenal insufficiency, *bpm* beats per minute, *HR* heart rate, *IVSTDd* interventricular septum thickness at diastole, *LAD* left atrial diameter, *LVEDd* left ventricular end-diastolic diameter, *LVEF* left ventricular ejection fraction, *MAP-D* mean arterial pressure-diastolic, *MAP-S* mean arterial pressure-systolic, *SVT* supraventricular tachycardia, *VPCs* ventricular premature complexes

**Table 3:** Clinical characteristics, echocardiography, 24 h Holter-ECG and 24 h-blood pressure monitoring findings in patients with primary (PAI) and secondary adrenal insufficiency (SAI)

	PAI patients (*n* = 11)	SAI patients (*n* = 6)
Clinical characteristics		
Age (years)	45.0 (18.0–65.0)	49.0 (42.0–67.0)
BMI (kg/m^2^)	24.4 (19.8–31.2)	26.1 (20.1–32.8)
Hypertension (%)	0	0
Diabetes mellitus (%)	1 (9)	0
Glycaemia (mg/dL)	77 (60–159)	84 (67–91)
Total cholesterol (mg/dL)	202 (152–284)	220 (177–321)
LDL-cholesterol (mg/dL)	119 (70–174)	143 (99–223)
Triglycerides (mg/dL)	114 (59–273)	179 (77–253)
s-Na (mmol/L)	139.0 (132.0–145.0)	140.5 (134.0–143.0)
s-K (mmol/L)	4.3 (4.0–5.1)	3.9 (3.6–10.9)
Echocardiography		
IVSTDd (mm)	8.5 (7.0–10.0)	9.2 (7.3–11.7)
LVEDd (mm)	45.9 (42.1–53.0)	49.8 (43.3–56.0)
LAD (mm)	34.0 (30.0–37.0)	38.0 (23.0–44.0)
LVEF (%)	64.5 (50.0–68.7)	64.5 (55.0–76.0)
Holter-ECG		
HR day (bpm)	82 (72–96)	74 (67–85)
HR night (bpm)	74 (65–90)	64 (58–80)
24 h-blood pressure		
MAP-S day (mmHg)	119 (96–138)	129 (106–132)
MAP-D day (mmHg)	72 (64–87)	79 (74–89)
MAP-S night (mmHg)	100 (94–124)	111 (91–143)
MAP-D night (mmHg)	63 (54–86)	67 (59–86)

Data are presented in median (range) or *n* (%)

*BMI* body mass index, *bpm* beats per minute, *HR* heart rate, *IVSTDd* interventricular septum thickness at diastole, *LAD* left atrial diameter, *LVEDd* left ventricular end-diastolic diameter, *LVEF* left ventricular ejection fraction, *MAP-D* mean arterial pressure-diastolic, *MAP-S* mean arterial pressure-systolic, *S-Na* serum Natrium level, *s-K* serum Kalium level

A significantly lower nocturnal blood pressure was found in AI patients compared with controls (Table 2; Fig. 2b). Particularly, the mean nocturnal systolic blood pressure (MAP-S night) was 108 ± 15 mmHg in patients and 117 ± 8 mmHg in controls (*P* = 0.038), and the mean diastolic blood pressure (MAP-D night) was 65 ± 9 vs. 73 ± 7 mmHg (*P* = 0.008). Conversely, no difference was recorded in mean blood pressure during daytime (Table 2; Fig. 2b). When the sub-group of patients with primary AI (*n* = 11) was compared to controls, a significantly lower mean diurnal systolic blood pressure was recorded (Supplementary Table 1). Median diurnal and nocturnal blood pressure in the sub-groups of patients with primary AI (*n* = 11) and secondary AI (*n* = 6) are shown in Table 3.

Holter-ECG analyses displayed similar HR in patients and controls, as was the number of VPCs. Two patients and two controls presented with asymptomatic SVT whereas no episode of VT was recorded (Table 2).

### Effects of switch from conventional GC replacement to OD-HC

**Fig. 3:**
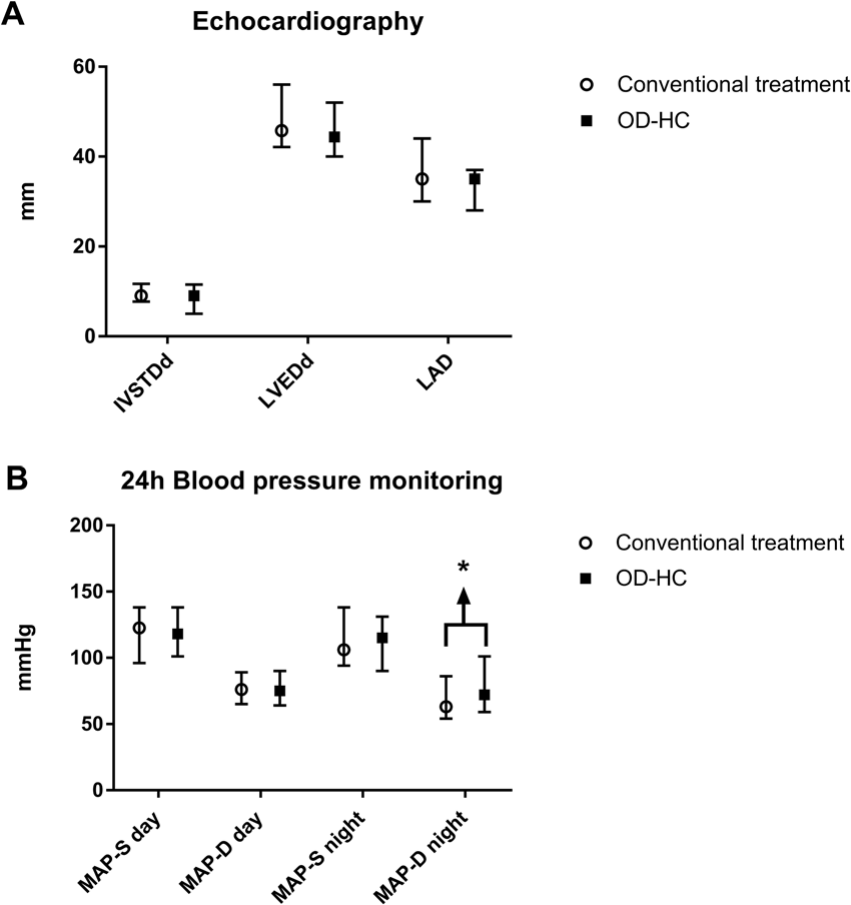
Echocardiographic and 24 h ambulatory blood pressure monitoring findings in patients with adrenal insufficiency before and after switching to once-daily modified-release hydrocortisone. IVSTDd interventricular septum thickness at diastole, LAD Left atrial diameter, LVDEDd left ventricle end-diastolic diameter, MAP-S mean systolic blood pressure, MAP-D mean diastolic blood pressure, OD-HC once-daily modified-release hydrocortisone. *, *P* < 0.05

Out of 17 patients with AI, 10 (7 patients with primary and 3 with secondary AI) were switched to an equivalent dose of OD-HC. After a 6-month period on stable treatment, total and LDL-cholesterol decreased whereas fasting plasma glucose was unaffected (Table 4). An increase in diastolic nocturnal blood pressure was observed after the switch to OD-HC [72 (59–101) vs. 63.0 (54–86) mmHg; *P* = 0.028], reaching a similar level to the healthy controls (Table 4; Fig. 3). However, diurnal blood pressure as well as systolic nocturnal blood pressure did not differ significantly. No significant changes were observed in echocardiographic and Holter-ECG parameters following the switch.

**Table 4:** Metabolic status, echocardiographic, 24 h Holter-ECG and 24 h-blood pressure monitoring findings in patients with adrenal insufficiency on conventional treatment and 6 months after switching to once daily modified release hydrocortisone (OD-HC)

	Conventional treatment (*n* = 10)	OD-HC treatment (*n* = 10)	Controls (*n* = 10)	P^*^	*P*^#^
Metabolic status					
Glycaemia (mg/dL)	81 (67–159)	83 (72–106)	83 (63–102)	0.244	0.953
Total cholesterol (mg/dL)	233 (152–321)	205 (152–241)	192 (162–230)	0.028	0.678
LDL-cholesterol (mg/dL)	135 (70–223)	109 (65–158)	125 (98–175)	0.028	0.139
Triglycerides (mg/dL)	111 (59–214)	92 (43–187)	146 (112–198)	0.176	0.066
Echocardiography					
IVSTDd (mm)	9.0 (7.7–11.7)	9.0 (5.0–11.5)	9.0 (8.0–10.0)	0.461	0.496
LVEDd (mm)	45.8 (42.1–56.0)	44.4 (40.0–52.0)	49.5 (48.0–55.0)	0.123	0.008
LAD (mm)	35.0 (30–44.0)	35.0 (28.0–37.0)	35.5 (33.0–41.0)	0.194	0.176
LVEF (%)	60.0 (50.0–68.3)	62.4 (50.6–70.0)	56.0 (50.0–64.0)	0.674	0.327
24 h-blood pressure					
MAP-S day (mmHg)	123 (96–138)	118 (101–138)	126 (108–135)	0.342	0.141
MAP-D day (mmHg)	76 (65–89)	75 (64–90)	76 (63–86)	0.374	0.398
MAP-S night (mmHg)	106 (94–138)	115 (90–131)	122 (103–127)	0.678	0.086
MAP-D night (mmHg)	63 (54–86)	72 (59–101)	71 (60–82)	0.028	0.674
Holter-ECG					
HR day (bpm)	81 (73–86)	81 (72–96)	81 (66–89)	0.400	0.646
HR night (bpm)	75 (58–90)	72 (55–86)	71 (48–79)	0.833	0.362

Data are presented in median (range). No cases of supraventricular tachycardia and ventricular tachycardia were recorded

*bpm* beats per minute, *HR* heart rate, *bpm* beats per minute, *IVSTDd* interventricular septum thickness at diastole, *LAD* left atrial diameter, *LVEDd* left ventricular end-diastolic diameter, *LVEF* left ventricular ejection fraction, *MAP-D* mean arterial pressure-diastolic, *MAP-S* mean arterial pressure-systolic

P* Conventional treatment vs. OD-HC treatment; P# OD-HC treatment vs. Controls

## Discussion

In the present study, echocardiographic abnormalities were recorded in patients with AI on replacement therapy with cortisone acetate twice or thrice daily compared with matched healthy controls. Despite both groups exhibited left ventricular function and architecture in the normal range, patients with AI displayed a smaller LVEDd as well as LAD than healthy subjects, suggesting a status of hypovolemia.

Patients with AI usually present with dehydration and hypovolemia at diagnosis. Hypotension as well as a loss of circadian variation in blood pressure has also been described [22, 23]. To our knowledge, however, no study investigating echocardiographic and other hemodynamic parameters in patients with AI on lifelong replacement therapy has been published so far.

Cardiac abnormalities, characterised by small left ventricular chamber size and high prevalence of mitral valve prolapse, were recorded in seven patients with primary AI at diagnosis, and normalised after 4–8 months of adequate replacement therapy [21]. Somewhat in contrast with these findings, we observed that patients with AI, despite on a long-term stable GC substitution, displayed smaller left-hearth chambers, higher LVEF and lower nocturnal blood pressure than healthy subjects, suggesting that the replacement therapy did not completely restore the hemodynamic status of the patients.

Published data on myocardial function in patients with AI are scanty and somewhat controversial, showing a left ventricular dysfunction in some studies but not in others [24–26]. Reduced left ventricular size and pericardial effusion have recently been reported in female patients with untreated secondary AI [27]. Another study, including nine patients with primary AI, showed that a 48 h discontinuation of GC replacement therapy was associated to diastolic left ventricular dysfunction [28]. Interestingly, Knowlton et al. [29] described a high prevalence of heart failure in patients with primary AI followed for up to 30 years. In that study, seven of 22 subjects with primary AI developed heart failure that was not related to their replacement regimens [29]. In our study, on the contrary, no patient had clinical or echocardiographic features consistent with impaired left ventricular systolic function.

According to several pre-clinical and clinical studies, cardiac ultrasound has an important role in the assessment of fluid status by providing information on ventricular diastolic and systolic function and loading condition [30, 31]. Short-term dehydration has been shown to lead to decreased left ventricular dimensions and development of mitral valve prolapse in healthy females [32]. After adequate rehydration, these echocardiographic features resolves. In this study, despite receiving a stable replacement therapy, patients with AI presented with echocardiographic signs of hypovolemia. This could be related to unphysiological GC replacement that fails to provide individualised cortisol exposure, as well as inadequate cortisol coverage during stress-related situation. In patients with primary AI, undertreatment with MCs should also be considered as a possible explanation [17].

Hypovolemia and hypotension are tightly related. Indeed, several evidences have showed that reduction of extracellular fluid in the body results in decreased mean arterial pressure [33–35]. In our study, a significant lower mean systolic and diastolic blood pressure during the night was recorded in AI patients in comparison with healthy controls. Consistently, Dunne et al. [26] have described lower blood pressure levels in 13 patients with hypopituitarism than in matched healthy controls. Available data suggest that AI patients have their lowest cortisol serum concentration in the early morning, at the time of highest endogenous cortisol levels in healthy subjects [36]. The current HC replacement regimen is unable to replicate the early morning peak resulting in a cortisol underexposure from ~3:00 a.m. until awaking time (e.g. time of the first HC dose), probably leading to nocturnal hypotension. An association between nocturnal hypotension and increased risk of CV events in both normotensive and hypertensive subjects has previously been reported [37] suggesting that this could be one of the mechanisms for increased CV mortality in AI.

The role of GC deficiency on cardiac electrical activity has not been fully elucidated. Preclinical studies in primary AI models have suggested that GC signalling plays an important role in the regulation of left ventricular function, whereas MC signalling has a preponderant effect in the modulation of cardiac electrical activity [38]. Interestingly, in that study, chronic GC deficiency was found to be related to left ventricular dysfunction and MC deficiency to arrhythmia [38]. Clinical data on cardiac electrical activity in patients with AI are scanty. Prolonged QT interval and life-threating arrhythmias, mainly due to electrolytes imbalance, have been reported in few case reports [39–41]. In our study, 24 h Holter-ECG was performed in patients with primary and secondary AI without recording any cardiac arrhythmias (Table 2). However, further studies with larger populations are needed to analyse this issue.

To our knowledge, this is the first study to analyse CV structure and function in patients with AI on conventional replacement therapy and after switching to OD-HC. After a 6-month period on stable treatment with OD-HC, lipid profile improved and diastolic nocturnal blood pressure increased to a similar level seen in healthy controls (Table 4; Fig. 3). These findings are consistent with recent evidences, showing that OD-HC provides a more physiological cortisol exposure, leading to improved cardio-metabolic profile [16, 42]. Indeed, the novel replacement treatment has an immediate-release coating and an extended-release core that better mimics the circadian rhythm of cortisol [15, 43, 44], that in turn could have beneficial effects on the circadian blood pressure profile. However, additional studies with larger sample size and longer follow-up are needed to further investigate this issue.

The main strengths of this study rely in the strict inclusion and exclusion criteria used to select the study population. However, its limitations are the relative short follow-up period (6 months after the switch to OD-HC) and the heterogeneity of the study group, including patients with both primary and secondary AI. Finally, a selection bias should be considered due to the limited number of subjects included.

In conclusion, our study shows echocardiographic signs of mild dehydration and a reduced nocturnal blood pressure in patients with AI on conventional therapy. These findings may suggest that hypovolemic status, typical of patients with AI at diagnosis, is not resolved by conventional GC replacement treatment. The switch to OD-HC seems to have a beneficial effect on blood pressure profile, but no effect on cardiac structure and function.

## Supplementary information

Supplementary Table 1
