# Supplementary material for: Patients with adrenal insufficiency have cardiovascular features associated with hypovolemia
Source: Endocrine. 2020 Aug 19;70(2):412–20. doi: 10.1007/s12020-020-02458-3 (PMC7581570; doi:10.1007/s12020-020-02458-3)
Supplement: Supplementary file 1 — Supplementary Table 1 [file 12020_2020_2458_MOESM1_ESM.docx]

| **Supplementary Table 1. Clinical characteristics, echocardiography, 24-hour Holter-ECG and 24h-blood pressure monitoring findings in patients with primary (PAI) and controls** | | | |
| --- | --- | --- | --- |
|  | **PAI patients**  **(n=11)** | **Controls**  **(n=11)** | ***P*** |
| ***Clinical characteristics*** | | | |
| Age (years) | 45.0 (18.0-65.0) | 51.0 (21.0-66.0) | 0.610 |
| BMI (kg/m^2^) | 24.4 (19.8-31.2) | 25.3 (22.6-29.4) | 1.000 |
| Glycaemia (mg/dL) | 77 (60-159) | 93 (71-106) | 0.154 |
| Total cholesterol (mg/dL) | 202 (152-284) | 193 (159-230) | 0.248 |
| LDL-cholesterol (mg/dL) | 119 (70-174) | 141 (123-175) | 0.041 |
| Triglycerides (mg/dL) | 114 (59-273) | 155 (109-221) | 0.062 |
| ***Echocardiography*** | | | |
| IVSTDd (mm) | 8.5 (7.0–10.0) | 9.0 (8.0–10.0) | 0.366 |
| LVEDd (mm) | 45.9 (42.1–53.0) | 52.0 (49.0-55.0) | 0.011 |
| LAD (mm) | 34.0 (30.0-37.0) | 38.0 (35.0-45.0) | 0.012 |
| LVEF (%) | 64.5 (50.0-68.7) | 53.0 (50.0-64.0) | 0.033 |
| ***Holter-ECG*** | | | |
| HR day (bpm) | 82 (72-96) | 70 (61-89) | 0.047 |
| HR night (bpm) | 74 (65-90) | 67 (48-82) | 0.114 |
| ***24h-blood pressure*** | | | |
| MAP-S day (mmHg) | 119 (96-138) | 122 (108-141) | 0.007 |
| MAP-D day (mmHg) | 72 (64-87) | 77 (63-90) | 0.108 |
| MAP-S night (mmHg) | 100 (94-124) | 115 (103-127) | 0.028 |
| MAP-D night (mmHg) | 63 (54-86) | 73 (60-84) | 0.018 |

Data is presented in median (range) or n (%). BMI, Body Mass Index; bpm, beats per minute; HR, heart rate; IVSTDd, Interventricular septum thickness at diastole; LAD, Left atrial diameter; LVEDd, Left ventricular end-diastolic diameter; LVEF, Left ventricular ejection fraction; MAP-D, Mean arterial pressure-diastolic; MAP-S, Mean arterial pressure-systolic; S-Na, serum Natrium level; s-K, serum Kalium level.
